# Oral hormone pregnancy tests and the risks of congenital malformations: a systematic review and meta-analysis

**DOI:** 10.12688/f1000research.16758.2

**Published:** 2019-01-29

**Authors:** Carl Heneghan, Jeffrey K. Aronson, Elizabeth Spencer, Bennett Holman, Kamal R. Mahtani, Rafael Perera, Igho Onakpoya

**Affiliations:** 1 Centre for Evidence-Based Medicine, Nuffield Department of Primary Care Health Sciences, University of Oxford, Oxford, UK; 2 Underwood International College, Yonsei University, Seoul, South Korea

**Keywords:** pregnancy; congenital malformations, hormones

## Abstract

**Background: **Oral hormone pregnancy tests (HPTs), such as Primodos, containing ethinylestradiol and high doses of norethisterone, were given to over a million women from 1958 to 1978, when Primodos was withdrawn from the market because of concerns about possible teratogenicity. We aimed to study the association between maternal exposure to oral HPTs and congenital malformations.

**Methods:** We have performed a systematic review and meta-analysis of case-control and cohort studies that included data from pregnant women and were exposed to oral HPTs within the estimated first three months of pregnancy, if compared with a relevant control group. We used random-effects meta-analysis and assessed the quality of each study using the Newcastle–Ottawa Scale for non-randomized studies.

**Results: **We found 16 case control studies and 10 prospective cohort studies, together including 71 330 women, of whom 4,209 were exposed to HPTs. Exposure to oral HPTs was associated with a 40% increased risk of all congenital malformations: pooled odds ratio (OR) = 1.40 (95% CI 1.18 to 1.66; P<0.0001; I ^2^ = 0%). Exposure to HPTs was associated with an increased risk of congenital heart malformations: pooled OR = 1.89 (95% CI 1.32 to 2.72; P = 0.0006; I ^2^=0%); nervous system malformations  OR = 2.98 (95% CI 1.32 to 6.76; P = 0.0109 I ^2^ = 78%); gastrointestinal malformations, OR = 4.50 (95% CI 0.63 to 32.20; P = 0.13; I ^2^ = 54%); musculoskeletal malformations, OR = 2.24 (95% CI 1.23 to 4.08; P= 0.009; I ^2^ = 0%); the VACTERL syndrome (Vertebral defects, Anal atresia, Cardiovascular anomalies, Tracheoesophageal fistula, Esophageal atresia, Renal anomalies, and Limb defects), OR = 7.47 (95% CI 2.92 to 19.07; P < 0.0001; I ^2^ = 0%).

**Conclusions:** This systematic review and meta-analysis shows that use of oral HPTs in pregnancy is associated with increased risks of congenital malformations.

## Introduction

Oral hormone pregnancy tests (HPTs), such as Primodos (known as Duogynon in Germany), were available as injections from 1950 and in tablet form in the UK from 1956 onwards, before the modern forms of urine pregnancy tests became available ^1^. Oral HPTs contained ethinylestradiol and large doses of norethisterone (synthetic forms of estrogen and progesterone respectively), the latter in much larger amounts than those included in current combined oral contraceptives (see Table 1). The test principle was that they would induce bleeding similar to menstruation in those who were not pregnant.

**Table 1.** Doses of ethinylestradiol and norethisterone in various formulations of contraceptive steroids, ordered by increasing dose of norethisterone.

Indication (oral formulation)	Ethinylestradiol dose	Norethisterone acetate dose
Progestogen-only contraception *	-	350 micrograms
Combined oral contraceptive (Loestrin-20)	20 micrograms	1000 micrograms
Combined oral contraceptive (Norimin)	35 micrograms	1000 micrograms
Biphasic combined oral contraceptive (BiNovum)	35 micrograms	500/1000 micrograms
Triphasic combined oral contraceptive (Synphase)	35 micrograms	500/1000/500 micrograms
Combined oral contraceptive (Loestrin-30)	30 micrograms	1500 micrograms
**Oral hormone pregnancy test (Primodos)**	**20 micrograms**	**10 milligrams**
In endometriosis, dysmenorrhoea, dysfunctional uterine bleeding, and menorrhagia, or to delay menstruation *	-	10–15 milligrams/day
Breast cancer *	-	40 milligrams/day

*Unbranded

In the UK more than a million women took HPTs ^2^. However, evidence that they should not be used in pregnant women because of a risk of fetal malformations ^3^ led the then Committee on Safety of Medicines in 1975 to conclude that a warning should be added to the Data Sheets, stating that HPTs should not be taken during pregnancy. ( Supplementary File 1) Warnings about HPTs in pregnancy first emerged in 1956: ^4^ accumulating concerns over an increased risk of malformations led to their withdrawal in a number of countries at different times. Norway cancelled the indication in pregnancy for HPTs in 1970; when the UK did so in 1978, the manufacturers of Primodos, Schering AG (taken over by Bayer AG in 2008), voluntarily stopped marketing the product; in Germany, Duogynon was taken off the market in 1981 ^1^.

Since Primodos was withdrawn, the discovery of previously confidential documents has led to renewed concerns about its potential to cause harm ^5^. In 2014, therefore, the Medicines and Healthcare products Regulatory Agency (MHRA) initiated a review, which was published in 2017 and reported that the evidence was insufficient, mixed, and too heterogeneous to support an association between oral HPTs and congenital malformations ^3^.

To date, there has been no systematic review and meta-analysis of oral HPTs, using all the available data, to assess the likelihood of an association. We have therefore performed a systematic review to obtain all relevant data on hormone pregnancy tests and congenital malformations, used meta-analytical tools to obtain summary estimates of the likelihood of an association, and assessed the potential biases in these estimates.

## Methods

### Data sources

Full details of our search strategy are provided in Supplementary File 2. We searched Medline, Embase, and Web of Science (which yielded German papers and conference abstracts) and searched for regulatory documents online, including the UK Government’s “Report of the Commission on Human Medicines’ Expert Working Group on Hormone Pregnancy Tests”, which includes the original Landesarchiv Berlin Files ^3^, and reference lists of retrieved studies from the start of the databases in 1946 to 20 February 2018.

We used the following search terms without date limits or language restrictions: (Primodos OR Duogynon OR "hormone pregnancy test" OR "sex hormones" OR "hormone administration" OR “norethisterone” OR “ethinylestradiol”) AND pregnancy AND (congenital OR malformations OR anomalies). Several comparable high-dose HPTs were available at the same time as Primodos; we performed additional searches for evidence relating to these (See Supplementary File 3 for List of HPTs included in evidence search).

### Study selection

We included observational studies of women who were or became pregnant during the study and were exposed to oral HPTs within the estimated first three months of pregnancy and compared them with a relevant control group. When a study was described in more than one publication, we chose the publication that contained the most comprehensive data as the primary publication. We excluded studies where the intervention was oral hormones taken for other reasons (e.g., oral contraception) and it was not possible to extract data on hormone pregnancy tests. We did not restrict the language of publication. We checked additional relevant data and extracted them from the secondary publications when necessary.

### Data extraction and risk of bias assessment

Two reviewers (CH and ES) applied inclusion and quality assessment criteria, compared results, and resolved discrepancies through discussion with the other authors. We used a review template to extract data on study type, numbers of pregnancies exposed and not exposed to oral HPTs, and types and numbers of outcomes. Where available, we extracted data about the women studied, including ascertainment of cases, age, parity, setting, exposure to other medications, and confounding variables. In case-control studies, if data were reported on more than one control group, we extracted data where possible for non-disease/non-abnormality controls, and combined control groups if necessary.

The primary outcome of interest was all major congenital malformations. We also categorized outcomes for the congenital anomaly in the offspring at any time into congenital cardiac, gastrointestinal, musculoskeletal, nervous system, and urogenital defects, and the VACTERL syndrome (Vertebral defects, Anal atresia, Cardiovascular anomalies, Tracheoesophageal fistula, Esophageal atresia, Renal anomalies, and Limb defects).

We assessed quality using the Newcastle–Ottawa Scale (NOS) for non-randomized studies included in systematic reviews ^6^. The scale assesses the selection of study groups (cases and controls), comparability of study groups, including cases and controls, and ascertainment of the outcome/exposure. Each positive criterion scores 1 point, except comparability, which scores up to 2 points. The maximum NOS score is 9, and we interpreted a score of 1 to 3 points as indicating a high risk of bias ^7^. To determine whether the study had controlled for the most important factors, we selected the items reported in the original paper and resolved disagreements through consensus, using a third author (IO). We examined whether there was a linear relation between methodological quality and study results, by plotting the odds ratios against the NOS scores, using Excel, and assessed the correlations of NOS scores with several confounding variables we collected ^8^.

### Data synthesis and statistical methods

We calculated study-specific odds ratios for outcomes and associated confidence intervals. We meta-analysed the data using a random-effects model. We assessed heterogeneity across studies using the I ^2^ statistic and publication bias using funnel plots ^9^. We performed a sensitivity analysis by removing single studies to judge the stability of the effect and to explore the effect on heterogeneity ^10^, and we described any sources of variation. We also judged robustness by removing studies of low quality from the analysis. To examine whether the observed heterogeneity could be explained by differences in the NOS score, we also performed meta-regression using the NOS score as the covariate against the log OR as weights for traditional meta-regression using Stata version 14.

We planned subgroup analyses for the timing of administration of HPTs in relation to pregnancy and organogenesis and study design (case-control versus cohort) using Cochran’s Q test. We used RevMan v.5.3 for all analyses, except for meta-regression, for which we used Stata version 14. RevMan and Stata estimate the effects of trials with zero events in one arm by adding a correction factor of 0.5 to each arm (trials with zero events in both arms are omitted). We performed a sensitivity analysis by removing studies with zero events from the analyses.

We followed the reporting guidelines of the Meta-Analysis of Observational Studies in Epidemiology (MOOSE). A completed checklist is available as Supplementary File 4 ^11^

### Patient involvement

Members of the Association for Children Damaged by HPTs were involved in the original discussions of this review and provided input to the outcome choices, the search, the location of study articles, and translations. We plan to present the study findings to relevant patient groups and make available lay interpretations.

## Results

### Description of included studies

**Figure 1.**
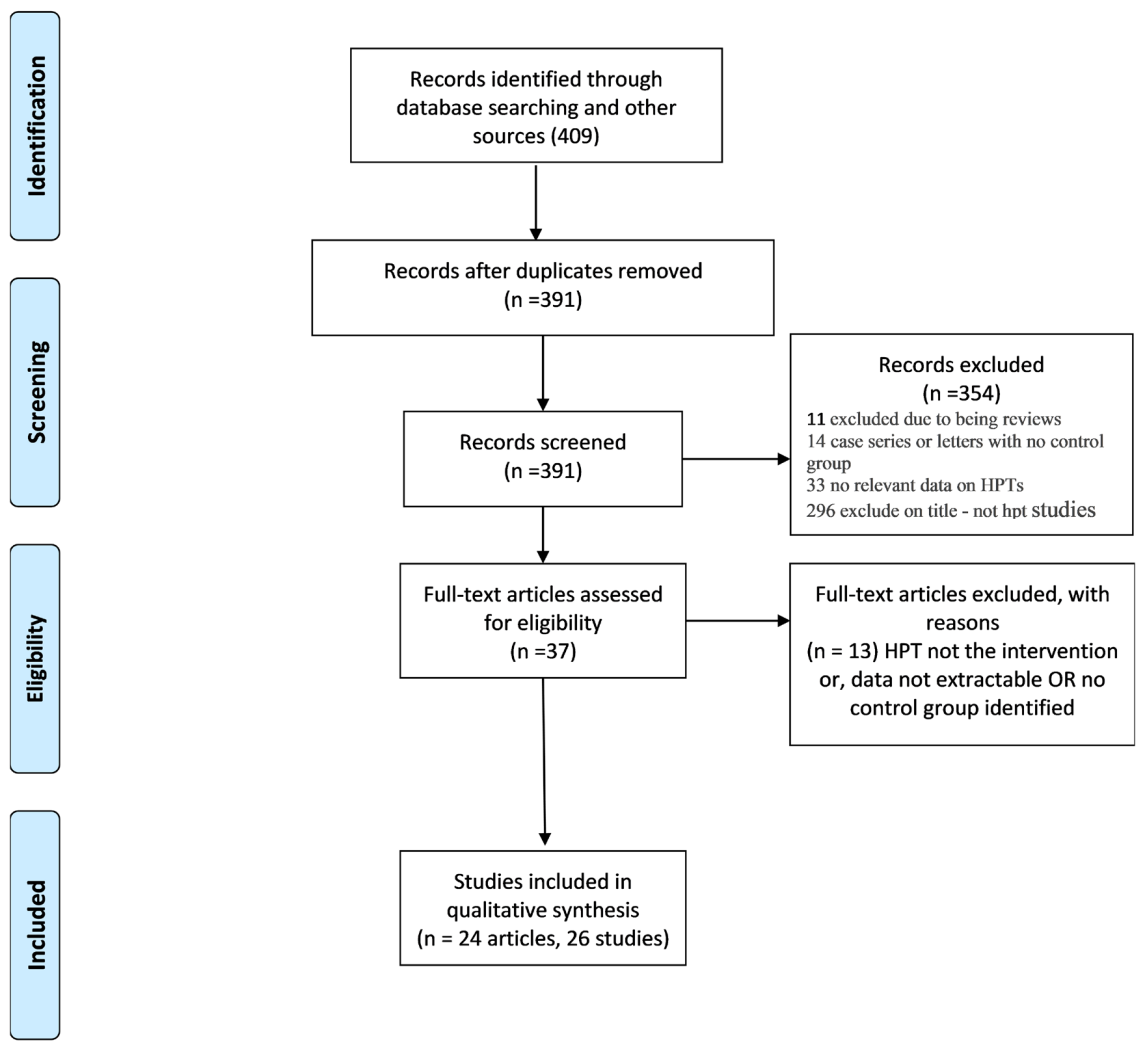
Study flow diagram showing inclusion of relevant studies.

We retrieved 409 items for screening. After title and abstract screening and removal of duplicates (n = 18), we excluded 354 records as not being relevant to the aim of the review. We assessed the full texts of 37 articles and identified 24 articles for inclusion. Figure 1 shows the PRISMA flow diagram for the inclusion of studies.

The 24 included articles reported on 26 studies (16 case-control studies and ten prospective cohort studies); one article [Nora 78] included two case-control studies and one prospective study. We found no randomized controlled trials. Of these articles, two were unpublished reports (see Supplementary File 5 for full references). The studies included 71,330 women. The case-control studies included 28,761 mothers, 594 of whom were exposed to HPTs; the cohort studies included 42,569 mothers and 3,615 exposures to HPTs. The studies were published between 1972 and 2014, and all were performed either in Europe or the USA. They mostly recruited women and their infants at maternity centres or hospital paediatrics wards.

The choices of controls in the case-control studies varied; they included, at one extreme, healthy infants born on a date close to the case infants and, at the other extreme, infants with malformations other than those under investigation. Among the prospective cohort studies, the populations tended to be women recruited at antenatal clinics or birth centres (See Table 2. Characteristics of included studies).

**Table 2.** Characteristics of included studies.

Study ID	Study population	Setting	Confounding variables collected	Information on controls including matching criteria in case-control studies	Outcomes reported
Case-control studies					
Ferencz 1980	Mothers of 110 infants with conotruncal abnormalities of the heart, born 1972–75.	Hospitals served by the Maryland State Intensive Care Neonatal Program, USA.	Maternal health (hospitalisations, illnesses, treatments); past reproductive history; index pregnancy factors including contraception used previously, fertility treatments, symptoms, illnesses and medications during pregnancy including hormones; smoking; alcohol intake; occupational history of mother and father; exposure of mother to fumes, paints and insecticides; family history including history of congenital abnormalities in previous children or in close relatives.	For each case, three unaffected controls were chosen from the birth population: two matched on eight characteristics related to the likelihood of hormone-taking (race, maternal age, parity, foetal losses, gestational age, delivery mode, time of prenatal registration, private service), and one also on the infant’s sex and birthweight; the third control was chosen at random.	Congenital heart disease (Conotruncal malformations of the heart)
Gal 1972	100 mothers of infants with spina bifida, and controls	Hospital in London, UK, for cases; unclear where controls were recruited from	Age, parity, reproductive history, illnesses, illegitimacy, bleeding	Controls matched for week of baby's birth; age of mother (5-year bands), reproductive history, course of pregnancy, sex of baby.	Spina bifida
Greenberg 1977	Cases identified via OPCS and matched controls identified from general practices of the cases.	General practices in the UK	Antenatal, personal, and family history and drugs prescribed during the first trimester.	Controls: babies born within 3 months of and based at the same general practice as matched cases. Antenatal, personal, and family history and drugs prescribed during the first trimester.	Neural tube defects, oral clefts, limb malformations and other non-minor abnormalities
Janerich 1974	108 cases of congenital limb defects and 108 unaffected controls	New York State, USA	Age, parity, race	Controls matched on birth date, mother's race and age +/- 2 years; and by default, due to adjacent records for cases and controls these matched well on county of residence of the mothers.	Congenital limb defects
Janerich 1977	104 cases with birth certificate mentioning CHD, 104 matched controls	New York State, USA	Age, country of residence, date of birth, race, medications, infections	From adjacent birth record matched by mother’s age, county of residence, date of birth, race	Congenital heart disease
Lammer 1986	1,091 mothers of infants with abnormalities born 1 July 190 to 20 June 1979, (21% not completed data collection)	Population register	Race, maternal education, family history, socio-economic status, parity, previous foetal loss	Control group was composed of infants with malformations other than the one under investigation. e.g. for spina bifida, controls were those with non-spina bifida abnormalities.	Major malformations, including anencephaly, spina bifida, cleft lip, cleft palate, Down syndrome, oesophageal atresia, small bowel atresia, rectal anal atresia, anterior abdominal wall defects, diaphragmatic hernia, limb reduction.
Laurence 1971	1968-1970, UK	3 hospital birth centres in the UK	Non-reported	In London the controls were the next baby with no abnormality born in the same hospital; in Exeter, control mothers were matched for area of birth, parity and month of conception; in Wales the control mothers were those who had had one baby with spina bifida or anencephaly and had a subsequent unaffected birth during the study period; these last were not matched individually.	Spina bifida and anencephaly
Levy 1973	76 cases, 76 controls	Hospital, Montreal, Canada	Non-reported	Controls were infants with Mendelian disorders, matched for date of birth.	Congenital heart defects (transposition of the great vessels)
Nora 1975	15 patients with multiple congenital anomalies. 30 controls (15 with chromosomal anomalies, 15 with functional heart murmurs)	University of Colorado Medical Center, Denver, and affiliated hospitals, USA	Age, race, socioeconomic status, area of residence	Matched for age. 15 controls had chromosomal abnormalities, 15 had functional heart murmurs	VACTERL
Nora 1978 case control 1	32 patients with VACTERL, 60 controls	Hospital	Age, date of birth, sex, gestational age, race, socioeconomic levels, areas of residences, parity	Matched as closely as possible for age, date of birth, sex, gestational age, race, socioeconomic levels, areas of residences, parity	VACTERL
Nora 1978 case control 2 and 3	236 patients with full variety of cardiac lesions, 412 controls with known single mutant gene and chromosomal disorders	Hospital	Sex, race, approximate date of birth, area of residence	Matching was for sex, race, approximate date of birth, area of residence	Congenital heart disease (congenital heart lesions)
Polednak 1983	99 singleton male births with hypospadias and 99 matched controls	New York State, USA	Parity, maternal age, race, area of residence	Most adjacent birth date, matched for maternal age, race, area of residence	Hypospadias
Rothman 1979	390 cases, 1,254 controls. HPTS: 14/388 cases vs 35/1246 controls	State care service for congenital heart disease	Parity, mother's education level, insulin use, alcohol, tobacco	Controls: births within same 3 years of the study period; 1,254 respondents from contacts to births selected randomly from the birth register.	Congenital heart disease
Sainz 1987	244 cases identified via the national collaboration of 42 hospitals registering congenital abnormalities between April 1976 to Sept 1984	Spanish register of congenital abnormalities within 42 participating hospitals	Sex, data and place of birth	Controls: unaffected births at same hospital, matched on sex, date of birth.	Spina bifida and anencephaly
Tummler 2014	296 cases, 3,676 infants with abnormalities	Data from the Malformation Monitoring Centre Saxony-Anhalt, Germany.	Non-reported	No information on matching	Congenital bladder exstrophy
Cohort studies					
Fleming 1978	RCGP Outcomes of Pregnancy study 1975: 9,000 women; from this was selected a random sample of 500 pregnancies proceeding to normal outcomes	General practices, UK	Non-reported		Any malformation
Goujard 1979	3,379 women pregnant and attending gynaecology clinics between 1975 to 1977	Obstetrics and gynaecology centres, Paris and Lille, France	Information on current pregnancies including symptoms and medications taken; previous pregnancies and general health backgrounds.		Congenital malformations, also congenital heart defects, skeletal anomalies, microencephaly.
Hadjigeorgiou 1982	Retrospective cohort, Alexandra Maternity Hospital Greece, births 1975-77. 15,535 live births, 559 exposed to sex hormones of which 112 (20%) exposed to HPTs, 14,976 no hormones, congenital heart disease studied confirmed by cardiologist & lab tests. Diseases and medication reported at admission prior to birth.	Hospital birth centre	Cytomegalovirus, infection, toxoplasmosis, hepatitis, syphilis, rubella, teratogenic drugs		Congenital heart disease
Haller 1974	3588 pregnant women, recruited Oct 1969 to April 1972, University Hospital Göttingen; 617 (17.2 %) with hormonal pregnancy test	Hospital birth centre	Non-reported		Congenital malformations
Kullander 1976	6,376 pregnancies, Malmo, 1963-5, resulting in 5,753 live births, 5,002/5,753 no abnormality, 751/5,753 with abnormality. 156 women took Primodos.	Sweden	Major and minor disease; the woman's age, parity, marital status, and social class. Birth weight, placental weight.		Major and minor malformations
Meire 1978	500 mothers consecutive births in 3 hospitals in Bruges, Belgium, 20 had taken HPTs.	Hospital birth centres	Non-reported		Oesophageal atresia
Michaelis 1983	13,643 pregnancies	Antenatal clinics, Germany	Detailed general and gynaecological history, drug intake, exposure to chemical agents, daily workload, intercurrent diseases, accidents, surgical operations and other factors.		Major malformations
Roussel 1968	Pregnancies 1966 to 1967	General practices, UK	NR		Central nervous system malformations including anencephaly, hydrocephaly, microcephaly, meningomyelocele, myelocele, spina bifida.
Rumeau- Rouquette 1978	1963-69, recruitment in 12 gynaecology clinics in Paris; 12,764 women gave birth to 12,895 children in hospitals participating in study; controls were mothers of unaffected infants selected at random among women questioned in same hospital	Hospital birth centres, France	Medical history, course of pregnancy, infectious diseases, inoculations, reproductive history, social and occupational category, use of alcohol, tobacco		Congenital malformations
Torfs 1981	19,906 full term pregnancies, 227 of which exposed to HPTs.	Hospital birth centre	Age, medical and reproductive history, socio- economic information, ethnicity		Severe congenital anomalies including congenital heart defects, neuroblastoma, cleft lip and limb reduction; non-severe congenital anomalies e.g. hypospadias of the first degree, congenital dis-location of the hip, polydactyly.

### Quality assessment of included studies

Of the 26 included studies, three were assigned a NOS score of 3 or below and were therefore judged as being at high risk of bias. One was a case-control study (Laurence 1971, a published abstract as a letter) and two were cohort studies (Fleming 1978 and Haller 1974, both unpublished). The NOS scores ranged from 2 to 9 (median 5). Twelve of the 26 included studies scored 7 to 9 and were judged to be at low risk of bias (see Table 3 of NOS scores in the data files). Item 5 of the NOS score addresses comparability of cases and controls based on design or analysis. Of the 16 case control studies, 12 controlled for the most important factor (item 5a) and nine controlled for important additional factors (item 5b). Of the ten cohort studies, six controlled for the most important factor (item 5a) and four controlled for important additional factors (item 5b). The mean Newcastle–Ottawa scale score was 6.1, indicating an overall moderate risk of bias. Table 2 also shows that seven studies did not report the confounding variables collected (Laurence 1971; Levy 1973; Tummler 2014; Fleming 1978; Haller 1974; Moire 1978; Rousel 1968). NOS scores correlated with the increasing number of confounding variables collected (r = 0.83). Supplementary File 6 shows the funnel plots for all congenital malformations and congenital heart disease; because of inadequate numbers of included studies, we did not use more advanced statistical methods to assess publication bias.

**Table 3.** Newcastle-Ottawa scale scores for included studies.

Newcastle–Ottawa scale case-control studies										
					Comparability of cases and controls on the basis of the design or analysis					
Study ID	Is the case definition adequate?	Are the cases representative?	Selection of controls adequate	Definition of controls adequate	a) Study controls for the most important factor	b) Study controls for important additional factors	Ascertainment of exposure adequate	Same method of ascertainment for cases and controls	Non-response rate adequate	Total score /9
Ferencz 1980	yes	yes	yes	yes	yes	yes	yes	yes	yes	9
Gal 1972	unclear	unclear	yes	yes	yes	yes	yes	yes	unclear	6
Greenberg 1977	yes	yes	yes	yes	yes	yes	unclear	yes	yes	8
Janerich 1974	no	yes	yes	no	yes	no	yes	yes	unclear	5
Janerich 1977	yes	yes	unclear	yes	yes	yes	yes	unclear	yes	7
Hellstrom 1976	yes	unclear	no	yes	yes	no	unclear	yes	unclear	4
Lammer 1986	yes	yes	unclear	yes	yes	yes	yes	yes	no	7
Laurence 1971	yes	unclear	unclear	yes	no	unclear	unclear	unclear	unclear	2
Levy 1973	yes	yes	no	yes	yes	no	unclear	unclear	unclear	4
Nora 1975	yes	yes	yes	yes	yes	no	yes	yes	yes	8
Nora 1978 case control 1	yes	yes	yes	no	yes	yes	yes	yes	yes	8
Nora 1978 case control 2 and 3	yes	yes	yes	no	yes	yes	yes	yes	unclear	7
Polednak 1983	yes	yes	yes	yes	yes	no	unclear	yes	unclear	6
Rothman 1979	yes	yes	no	no	no	yes	yes	yes	unclear	5
Sainz 1987	unclear	yes	yes	unclear	yes	yes	unclear	yes	unclear	5
Tummler 2014	yes	no	no	no	no	no	no	yes	yes	3
Newcastle–Ottawa scale cohort studies										
					Comparability of cases and controls on the basis of the design or analysis					
	Are the participants representative of the exposed cohort?	Is the non- exposed cohort similar to the exposed?	Is the ascertainment of exposure accurate?	Is there evidence that the outcome of interest was not present at start of study?	a) Study controls for the most important factor	b) Study controls for important additional factors	Was the assessment of outcome adequate?	Was follow-up long enough for outcomes to occur?	Was there adequacy of follow up of cohorts?	Total score /9
Fleming 1978	yes	unclear	unclear	yes	unclear	unclear	unclear	yes	unclear	3
Goujard 1979	yes	yes	unclear	yes	yes	no	unclear	yes	yes	6
Hadjigeorgiou 1982	yes	yes	yes	yes	no	yes	yes	yes	unclear	7
Haller 1974	unclear	unclear	unclear	yes	no	no	unclear	yes	unclear	2
Kullander 1976	yes	yes	yes	yes	yes	unclear	yes	yes	yes	8
Meire 1978	yes	no	yes	yes	yes	no	unclear	yes	yes	6
Michaelis 1983	yes	yes	yes	yes	yes	yes	yes	yes	yes	9
Roussel 1968	yes	yes	yes	yes	no	no	unclear	yes	yes	6
Rumeau- Rouquette 1978	yes	unclear	yes	yes	yes	yes	yes	yes	no	7
Torfs 1981	yes	yes	yes	yes	yes	yes	yes	yes	yes	9

### Association of exposure to HPT with the risks of malformations

**Figure 2.**
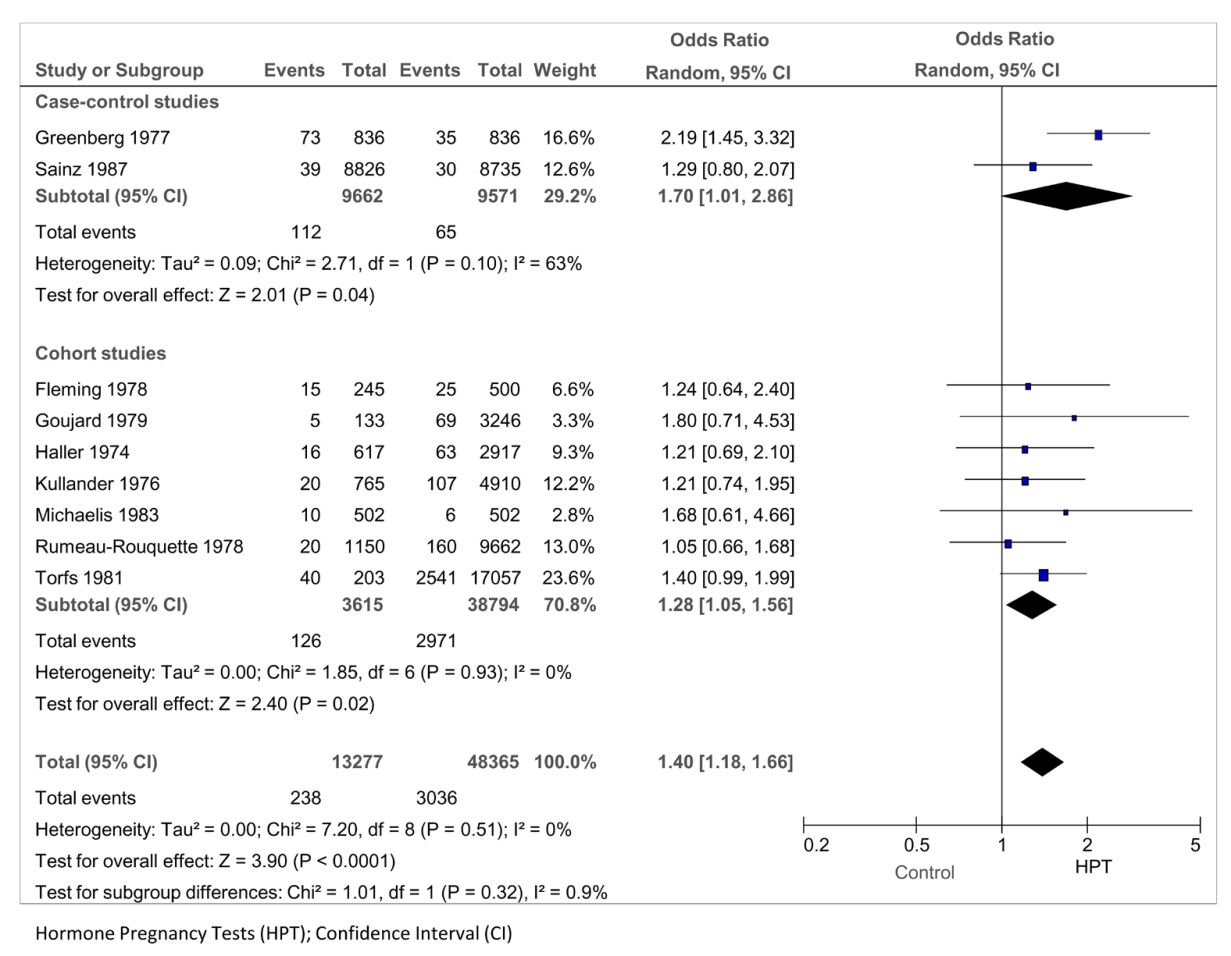
Association of exposure to oral HPTs in pregnancy with all malformations in the offspring.

Nine studies, including 61,642 mothers of infants and 3,274 exposed to HPTs, examined the association in pregnancy with all congenital malformations. Two were case-control studies (Greenberg 1977; Sainz 1987) and seven were cohort studies (Fleming 1987; Goujard 1979; Haller 1974; Kullander 1976; Michaelis 1983; Rumeau-Rouquette 1978; Torfs 1981) ( Figure 2). Exposure to oral HPTs was associated with a 40% increased risk of all congenital malformations: pooled odds ratio (OR) = 1.40 (95% CI 1.18 to 1.66; P < 0.0001; I ^2 ^= 0%). For the two case-control studies only, pooled OR = 1.70 (95% CI 1.01 to 2.86; P = 0.04; I ^2^ = 63%) and for the seven cohort studies, pooled OR = 1.28 (95% CI 1.05 to 1.56; P = 0.02; I ^2^ = 0%). The test for subgroup differences was not significant (P = 0.32). In a post-hoc sensitivity analysis, removing the studies that collected no confounding variables (Haller 74 and Fleming 78, both of low quality) did not affect the significance of the result (OR 1.44; 95% CI 1.18 to 1.75; P = 0.0004, I ^2^ = 11%). The meta-regression showed no association between total NOS score and increased risk (P = 0.51).

**Figure 3.**
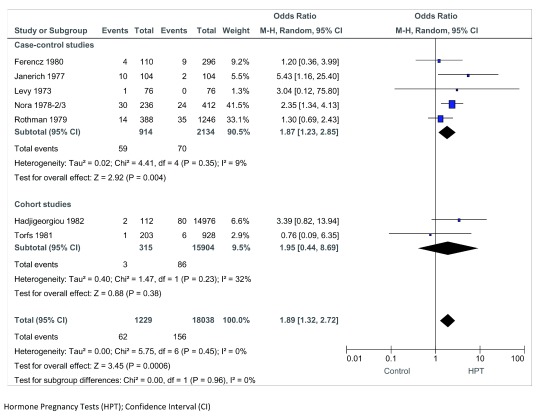
Association of exposure to oral hormone pregnancy tests (HPTs) in pregnancy with congenital heart disease in the offspring.

Seven studies, including 19,267 mothers of infants and 218 exposed to oral HPTs, analysed congenital heart malformations. Five were case-control studies (Ferencz 1980; Janerich 1977; Levy 1973; Nora 1978-2/3) and two were cohort studies (Hadjigeorgiou 1982; Torfs 1981) ( Figure 3). The pooled relative OR = 1.89 (95% CI 1.32 to 2.72; P = 0.0006; I ^2 ^= 0%).

In a post-hoc sensitivity analysis, removing one study that collected no confounding variables (Levy 73, a low-quality study) did not affect the significance of the result (OR = 1.88; 95% CI 1.25 to 2.85; P = 0.003, I ^2^ = 12%) For the five case-control studies only, the pooled OR = 1.87 (95% CI 1.23 to 2.85; P = 0.004; I ^2^ = 9%); for the two cohort studies the pooled OR = 1.95 (95% CI 0.44 to 8.69; P = 0.38; I ^2^ = 32%). The meta-regression was not significant (P = 0.94).

**Figure 4.**
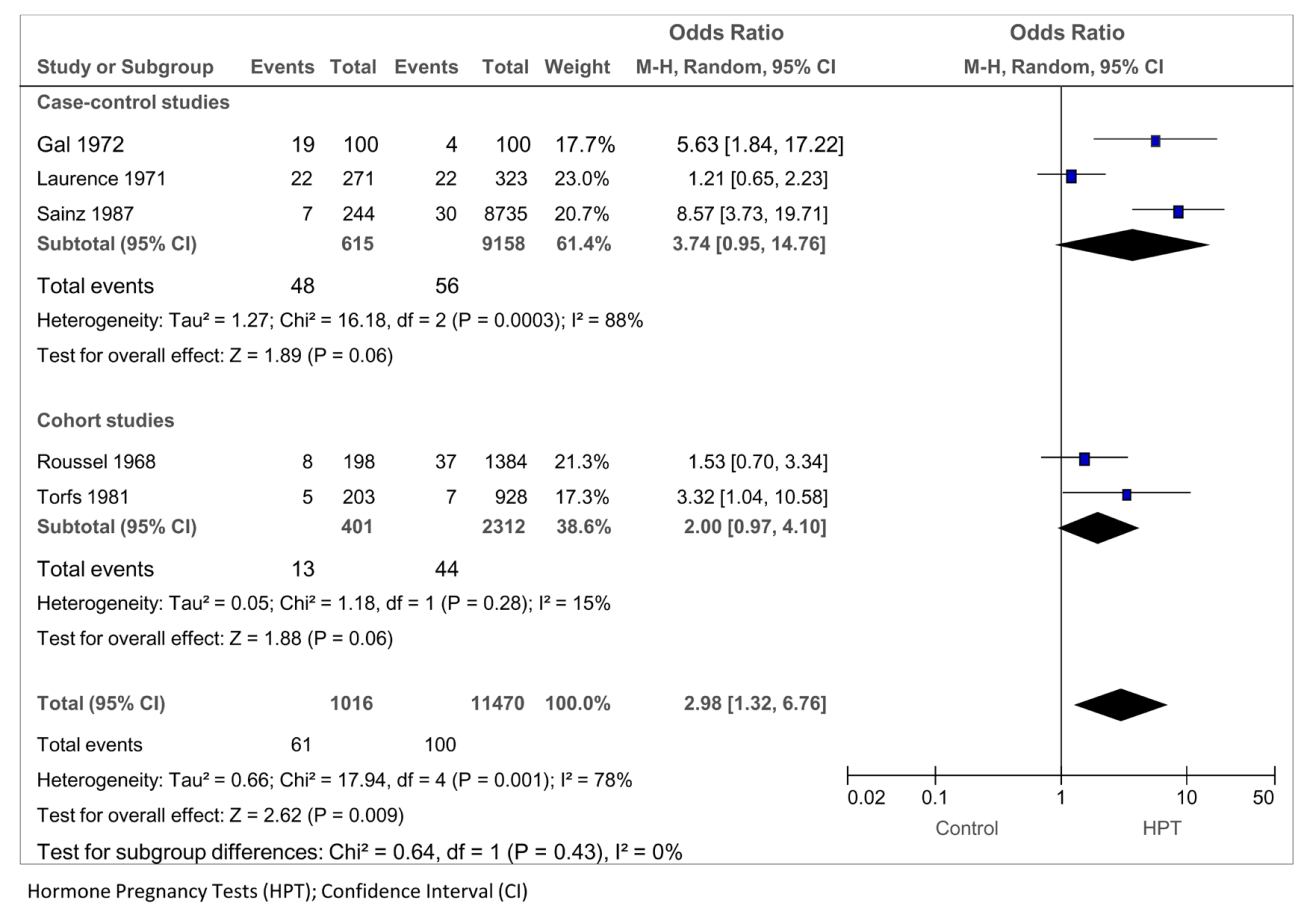
Association of exposure to oral hormone pregnancy tests (HPTs) in pregnancy and nervous system malformations in the offspring.

For the association between exposure to oral HPTs and nervous system malformations in the offspring, five studies provided data: three case-control studies (Gal 1972; Laurence 1971; Sainz 1987) and two cohort studies (Roussel 1968; Torfs 1981), including 12 486 mothers of infants and 127 exposed ( Figure 4). The pooled OR = 2.98 (95% CI 1.32 to 6.76; P = 0.009; I ^2 ^= 78%). In a post-hoc sensitivity analysis, removing the two studies that collected no confounding variables (Laurence 71; Roussel 68) did not affect the significance of the result and removed the heterogeneity (OR 6.04; 95% CI 3.33 to 10.78; P < 0.00001, I ^2^ = 0%).

**Figure 5.**
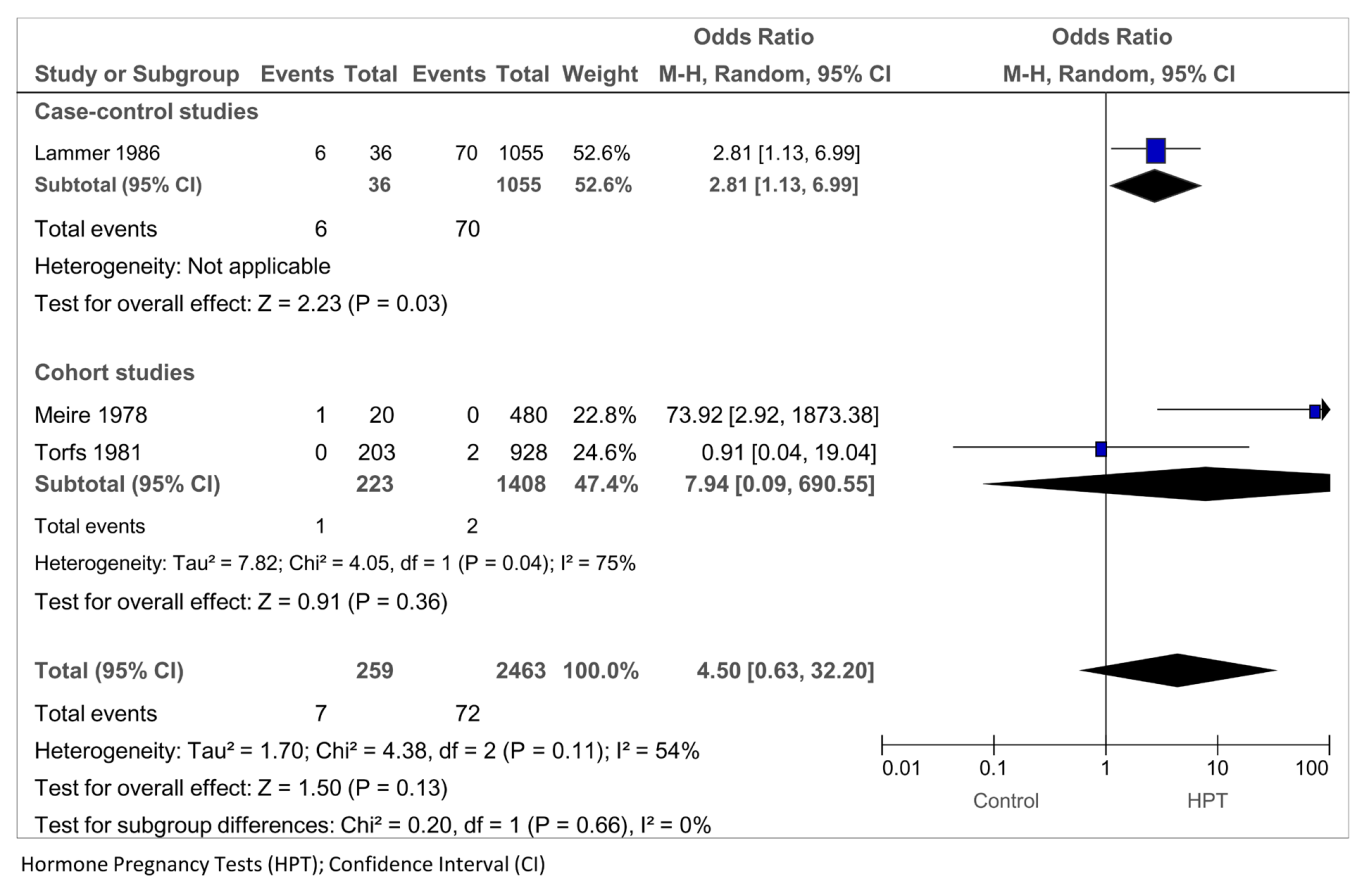
Association of exposure to oral hormone pregnancy tests (HPTs) in pregnancy and gastrointestinal malformations in the offspring.

**Figure 6.**
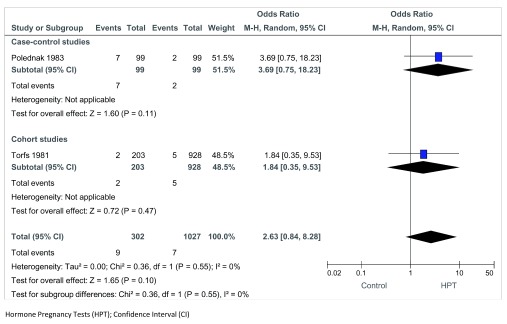
Association of exposure to oral hormone pregnancy tests (HPTs) in pregnancy and urogenital malformations in the offspring.

Gastrointestinal malformations and exposure to oral HPTs were reported in three studies: a case-control study (Lammer 1986) and two cohort studies (Meire 1978 and Torfs 1981), providing data on 2,722 mothers of infants, including 79 exposed to HPTs ( Figure 5). The pooled OR = 4.50 (95% CI 0.63 to 32.20; P = 0.13; I ^2 ^= 54%). One case-control study (Polednak 1983) and one cohort study (Torfs 1981) examined the relationship between exposure to oral HPTs in pregnancy and urogenital malformations: pooled OR = 2.63 (95% CI 0.84 to 8.28; P = 0.10; I ^2 ^= 0%) ( Figure 6).

**Figure 7.**
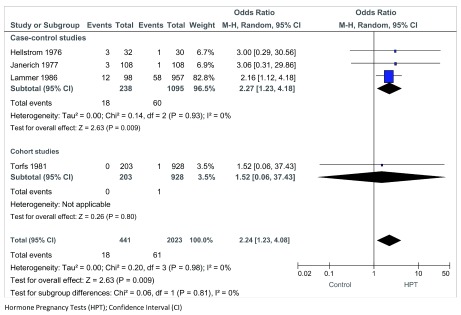
Association of exposure to oral hormone pregnancy tests (HPTs) in pregnancy and musculoskeletal malformations in the offspring.

**Figure 8.**
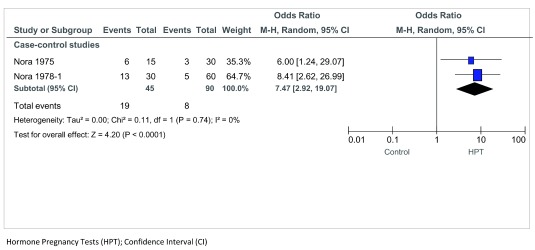
Association of exposure to oral hormone pregnancy tests (HPTs) in pregnancy with Vertebral defects, Anal atresia, Cardiovascular anomalies, Tracheoesophageal fistula, Esophageal atresia, Renal anomalies, and Limb defects (VACTERL) syndrome in the offspring.

A relation between the exposure to oral HPTs and musculoskeletal malformations was reported in three studies: three case-control studies (Hellstrom 1976; Janerich 1977; Lammer 1986) and one cohort study (Torfs 1981) ( Figure 7), based on 2,464 women, with 79 exposed to HPTs. The pooled OR = 2.24 (95% CI 1.23 to 4.08; P = 0.009; I ^2 ^= 0%). Removal of the zero study events (Torfs 1981) did not affect this result. The association of VACTERL with HPT exposure was reported in two case-control studies (Nora 1978-1 and Nora 1975), based on 135 women and infants and 27 exposed to HPTs; the OR was 7.57 (95% CI 2.92 to 19.07; P < 0.0001; I ^2 ^= 0%) ( Figure 8).

Study extraction sheet Click here for additional data file. Copyright: © 2019 Heneghan C et al. 2019 Data associated with the article are available under the terms of the Creative Commons Zero "No rights reserved" data waiver (CC0 1.0 Public domain dedication).

## Discussion

We found 24 articles containing 26 studies that reported the association between exposure to oral hormone pregnancy tests in mothers and malformations in their infants: 16 were case-control studies and ten were prospective cohort studies. The overall quality of the evidence, assessed by the Newcastle–Ottawa Scale, was moderate.

We found significant associations for all congenital malformations pooled and separately for congenital heart malformations, nervous system malformations, musculoskeletal malformations, and the VACTERL syndrome. Many of these pooled analyses had zero heterogeneity, and the direction of effect favoured the controls in 30 of the 32 analyses undertaken (Torfs 81 provided the only effect estimate favouring HPT exposure). The analyses were also robust to sensitivity analyses, and there was no relation between NOS score and increasing risk.

Based on the assumptions that a teratogenic effect of HPTs would be mediated by actions on estrogen and progestogen receptors, and that concentrations of ethinylestradiol and norethisterone in the fetus would be too low to have a significant effect on those receptors, it has been suggested that there is no mechanistic argument for teratogenicity ^3^. However, other unknown mechanisms might be at play. For example, Isabel Gal first reported concerns of malformations in the children of mothers exposed to HPTs in 1967 ^12^, pointing out that bleeding often occurred in pregnant women soon after exposure and suggesting that that would affect the “equilibrium” of the uterus. Between 5 and 11% of exposed women had bleeding, and the RCGP survey reported induced abortions in about 10% of women ^13^.

The drugs in Primodos were not tested for animal toxicity and teratogenicity at the time, which, although not unusual, meant that there was a gap in mechanistic understanding. A 2018 study showed that the components in Primodos are associated with dose-dependent and time-related damage in zebrafish embryos, and affect nerve outgrowth and blood vessel patterning in zebrafish ^12, 14^. Although it is difficult to compare drug actions between species, and evidence from animal studies is limited, the drugs accumulated in the zebrafish embryos, persisted for some time, and led to rapid embryonic damage ^12, 14^. In contrast, other animal studies have shown minimal effects on embryo development ^15^. There is also evidence that estradiol and progestogens increase the expression of mRNA for isoforms of vascular endothelial growth factor (VEGF) in Ishikawa cells from human endometrial adenocarcinoma ^16^.

### Strengths and weaknesses

Establishing causal associations in the absence of randomization can be difficult. However, the lack of randomized trials in our analysis should not be seen as a barrier to interpreting our findings. It would have been unethical to randomize individuals to drugs with known concerns, and randomization, like systematic reviews, was not the norm at the time. Furthermore, for questions about harms, the Oxford CEBM levels of evidence puts systematic reviews of case-control studies on a par with systematic reviews of randomized trials ^17^.

However, observational methods have limitations ^18^. First, interpretation can be affected by confounding factors. Although most of the studies in this review used matched controls, our analysis was based on raw data from the publications and did not adjust for confounders. Secondly, susceptibility bias can occur, as women with threatened abortions might be more likely to present and take the medication. Both of these problems can be mitigated by careful matching; 13 of the 16 studies controlled for the most important factor, item 5a on the NOS scale. Thirdly, the severity of malformations studied will have led to differing risk estimates across studies. Fourthly, inappropriate methods of ascertainment of the malformations and exposures could have introduced bias. Finally, incomplete and uneven reporting, along with publication bias (since it is likely that unreported studies exist) could introduce bias and alter the effect estimates.

The use of scoring systems to assess quality has been criticized. However, the NOS scale has been used widely in assessing the quality of non-randomized studies ^19– 24^. A NOS score between 0 and 9 has previously been used as a potential moderator in meta-regression ^25^, and has been recommended by the Cochrane Collaboration ^26^. A weakness of the NOS scale is the possible low agreement between assessors ^27^. This was particularly the case when authors had limited experience in doing systematic reviews, but training, even of novices, improves agreement ^19^.

The effects were also stable to sensitivity analyses, and changes in NOS score did not affect the risk estimates. The absence of subgroup differences between study designs for the risk estimates supports the robustness of the findings. We also tried to overcome publication bias by translation and assessment of unpublished data. The sample sizes in the studies for all congenital malformations, congenital heart disease, and nervous system malformations were sufficiently large to suggest that small unpublished studies would have little effect on the estimates unless they were highly heterogeneous. The analyses of gastrointestinal, urogenital, musculoskeletal, and VACTERL malformations were limited by their small sample sizes and low number of events: the interpretation of these effects should, therefore, be treated more cautiously. The significant effect observed for VACTERL should also be treated cautiously, as the confidence intervals for this effect were wide.

Our study has several strengths. We used standard systematic review methods, and by asking a focused question solely on exposure to HPTs, and excluding exposure to other hormones, we have been able to assess the heterogeneity of the effect estimates. However, as with any observational studies, there is always the possibility that an unknown confounder could be the cause of the observed difference. While such a possibility cannot be ruled out, the lack of heterogeneity means that such a confounder would potentially have to act in the same direction, despite many different confounders being collected and controlled for. Confounding factors with variable effects on the effect estimates would have probably led to a high degree of heterogeneity, which would have prevented pooling; this was not the case.

## Conclusion

**Figure 9.**
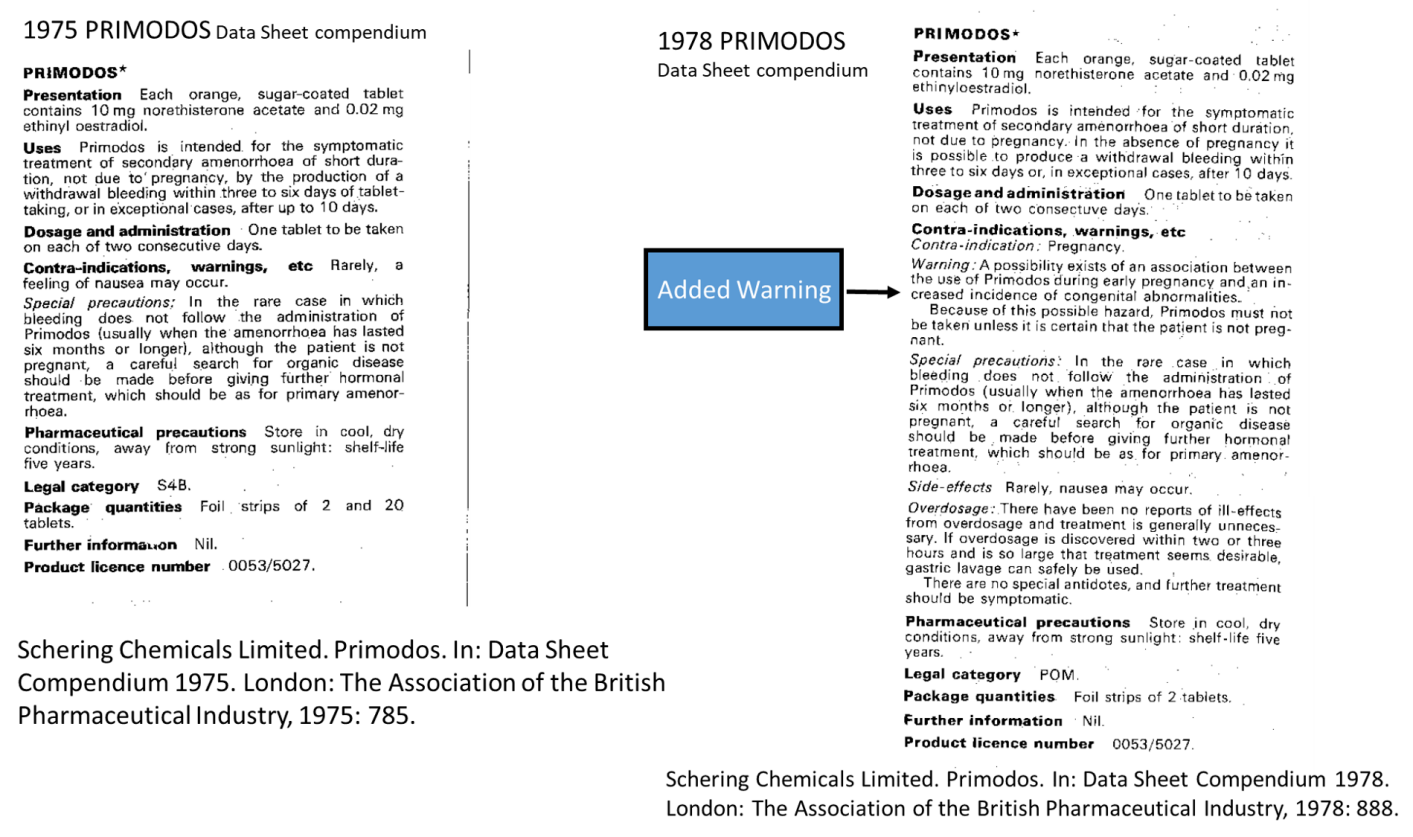
Primodos label 1975 and 1978.

Regulators were first made aware of the link between exposure to HPTs and congenital malformations in 1967. After 1975, the Primodos label was changed to state that the medication should not be used in pregnancy because of a risk of malformations (see Figure 9). The evidence of an association has previously been deemed weak, and previous litigation and reviews have been inconclusive. However, we believe that this systematic review shows an association of oral HPTs with congenital malformations.

Our results show the benefit of undertaking systematic reviews, a study type not in routine use when most of these studies were done. For example, only one study (Greenberg 1997) out of nine reported a significant effect for all congenital malformations; the pooled estimate was significant. Much of the discussion over the associations of HPTs with congenital malformations at the time these studies were published focused on the lack of significance of individual studies ^28^, although it was also recognized that the numbers involved were insufficient to reject the hypotheses ^29^.

## Declarations

### Data availability

**F1000Research: Dataset 1. Study extraction sheet,** https://dx.doi.org/10.5256/f1000research.16758.d233494 ^30^

## Supplementary material

**Supplementary File 1.** Committee on Safety of Medicines report 1975-colour pdf.

**Supplementary File 2.** Search Strategy.

**Supplementary File 3.** List of hormone pregnancy tests (HPTs) included in evidence search.

**Supplementary File 4.** Completed Meta-analysis Of Observational Studies in Epidemiology (MOOSE) checklist.

**Supplementary File 5.** Full references for included studies.

**Supplementary File 6.** Funnel Plots.
