# Supplementary material for: Oral hormone pregnancy tests and the risks of congenital malformations: a systematic review and meta-analysis
Source: F1000Res. 2019 Jan 29;7:1725. Originally published 2018 Oct 31. [Version 2] doi: 10.12688/f1000research.16758.2 (PMC6281024; doi:10.12688/f1000research.16758.2)
Supplement: Supplementary file 2 [file f1000research-7-19519-s0001.tgz › 1972e593-f6ed-462a-bb6d-dabd714daa65_Supplementary_FIle_1_CSM_1975-colour.pdf]

HORMONAL PREGNANCY TESTS:
A possible association with congenital abnormalities

COMMITTEE ON SAFETY OF MEDICINES

A number of studies have shown a possible association between taking mixtures of an oestrogen and a progestogen as a means of diagnosing pregnancy and an increased incidence of congenital abnormalities.

The Committee on Safety of Medicines wish to draw attention to these studies and to the preliminary results of their own case-control study. The early results suggest that a relatively greater proportion of mothers of abnormal babies had been tested in this way. A letter describing these preliminary results was published in the British Medical Journal on April 26 1975. (Greenberg, et al, ii, 191). The Committee will present their further conclusions later in the year, when their study is completed.

On the present evidence, the Committee believe that it is possible that the use of these preparations for the diagnosis of pregnancy could on occasion lead to abnormalities in the foetus. There are other means of diagnosing pregnancy which do not require the administration of hormones, and the Committee consider that in view of this possible hazard this method should not now normally be used.

As the data began to accumulate it was felt advisable to inform the companies known to be concerned and it was ascertained either that they had ceased to promote the products for this use, or that the product had been removed from the market. With this further evidence of this possible hazard, the Committee have advised the Health Departments that measures should be taken to ensure that this indication is not included in licences for such products and to require the insertion in all promotional literature of a warning about this possible hazard in pregnancy.

As far as is known the hormone preparations which have been, at some time, used or recommended for this purpose are:

Amenorone	Norlestrin	Paralut
Amenorone Forte	Norlutin A	Pregornot
Disecron	Norone	Primodos
Menstrogen	Orasecron	Secrodyl

Some of these products are no longer on the market, whilst others will continue to be marketed for the treatment of a variety of conditions in women who are not pregnant.

ADVERSE REACTIONS SERIES

NO 13
JUNE 1975

Committee on Safety of Medicines,
Finsbury Square House,
33/37A Finsbury Square,
LONDON EC2A 1PP.

Telephone: 01-638 6020
