# Supplementary material for: Oral hormone pregnancy tests and the risks of congenital malformations: a systematic review and meta-analysis
Source: F1000Res. 2019 Jan 29;7:1725. Originally published 2018 Oct 31. [Version 2] doi: 10.12688/f1000research.16758.2 (PMC6281024; doi:10.12688/f1000research.16758.2)
Supplement: Supplementary file 3 [file f1000research-7-19519-s0002.tgz › 6bac13aa-23e4-4ca5-aa4b-f37ed5d8036c_Supplementary_FIle_2_Search_strategy_.docx]

| 1 | Pregnancy Tests/ |  |
| --- | --- | --- |
| 2 | pregnancy test*.ti,ab. |  |
| 3 | 1 or 2 |  |
| 4 | Norethindrone/ |  |
| 5 | Ethinyl Estradiol/ |  |
| 6 | (norethindrone or norethisterone or ethinylnortestosterone or ethinyl estradiol or ethinylestradiol).ti,ab. |  |
| 7 | hormon*.ti. |  |
| 8 | 4 or 5 or 6 or 7 |  |
| 9 | 3 and 8 |  |
| 10 | (hormon* adj3 pregnancy test*).ti,ab. |  |
| 11 | (primodos or duogonyn).af. |  |
| 12 | 9 or 10 or 11 |  |
| 13 | exp animals/ not humans.sh. |  |
| 14 | 12 not 13 |  |
