# Supplementary material for: Oral hormone pregnancy tests and the risks of congenital malformations: a systematic review and meta-analysis
Source: F1000Res. 2019 Jan 29;7:1725. Originally published 2018 Oct 31. [Version 2] doi: 10.12688/f1000research.16758.2 (PMC6281024; doi:10.12688/f1000research.16758.2)
Supplement: Supplementary file 4 [file f1000research-7-19519-s0003.tgz › 99633850-ecee-4316-9761-d12ca9584cfc_Supplementary_File_3_List_of_HPTs_included_in_Search.docx]

**Appendix 2.** List of formulations for hormone pregnancy testing included in the evidence search

| **Brand name** | **Contents** | | **Date withdrawn from the UK market**  **(if marketed as a pregnancy test)** |
| --- | --- | --- | --- |
|  | **Estrogen** | **Progestogen** |  |
| Amenorone* | Ethinlyestradiol 10 micrograms | Ethisterone  10 milligrams | May 1977 |
| Amenorone Forte* | Ethinlyestradiol 50 micrograms | Ethisterone  50 milligrams | May 1977 |
| Disecron* | - | - | March 1969 |
| Duogynon | Estradiol benzoate 3 mg | Norethisterone 10 mg |  |
| Duphaston | – | Dydrogesterone 10 mg | [The 1975 Data Sheet does not mention diagnosis of pregnancy as an indication] |
| Estro-Prodial | - | - |  |
| Gestest | Ethinlyestradiol 50 micrograms | Norethisterone acetate 2.5 mg |  |
| Menstrogen* | Ethinlyestradiol 10 micrograms | Ethisterone  10 milligrams | March 1975 |
| Norlestrin* | Ethinlyestradiol 50 micrograms | Norethisterone acetate 2.5 mg |  |
| Norlutin-A* | – | Norethisterone 17β-acetate  2.5 milligrams | 1975 |
| Norone* | Mestranol 300 micrograms | Norethynodrel 20 milligrams | January 1969 |
| Orasecron* | Ethinlyestradiol 50 micrograms | Ethisterone  10 milligrams | June 1975 |
| Paralut* | Ethinlyestradiol 50 micrograms | Ethisterone  10 milligrams | Before 1971 |
| Paralut Forte | Ethinlyestradiol 100 micrograms | Ethisterone  50 milligrams |  |
| Pregornot* | Ethinlyestradiol 50 micrograms | Ethisterone  50 milligrams | Not known |
| Pro-Duosterone | Ethinlyestradiol 30 micrograms | Ethisterone  50 milligrams |  |
| Secrodyl* | Ethinlyestradiol 50 micrograms | Dimethisterone 10 milligrams | February 1975 |

Note: In the USA norethisterone (INN and BAN) is called norethindrone (USAN)

*These formulations were listed in the CSM’s yellow warning sheet in 1975 (Number 13 in its Adverse Reactions Series)

Sources: Pregnancy Testing. Hansard 198. https://api.parliament.uk/historic-hansard/written-answers/1978/apr/10/pregnancy-testing. Todd RG (ed). *Extra Pharmacopoeia*. *Martindale.* 25^th^ ed. London: The Pharmaceutical Press, 1967. Wade A (ed), Reynolds JEF (asst ed). *Martindale. The Extra Pharmacopoeia*. 27^th^ ed. London: The Pharmaceutical Press, 1977.
