# Supplementary material for: Oral hormone pregnancy tests and the risks of congenital malformations: a systematic review and meta-analysis
Source: F1000Res. 2019 Jan 29;7:1725. Originally published 2018 Oct 31. [Version 2] doi: 10.12688/f1000research.16758.2 (PMC6281024; doi:10.12688/f1000research.16758.2)
Supplement: Supplementary file 6 [file f1000research-7-19519-s0005.tgz › f433629f-6a6c-40fb-9cb7-a7680b4adab4_Supplementary_File_5_Full_references.docx]

**Appendix 3.** Full references for included studies

Ferencz C, Matanoski GM, Wilson PD, Rubin JD, Neill CA, Gutberlet R (1980) Maternal hormone therapy and congenital heart disease. Teratology 21(2): 225-239.

Gal I (1972) Risks and benefits of the use of hormonal pregnancy test tablets. Nature 240: 241-242

Greenberg, G, Inman WHW, Weatherall JAC, Adelstein AM, Haskey JC (1977) Maternal drug histories and congenital anomalies. BMJ 2: 853-856.

Hellstrom B, Lindsten J, Nilsson K (1976) Prenatal sex-hormone exposure and congenital limb reduction defects. Lancet 2:372-3.

Lammer EJ, Cordero JF (1986) Exogenous sex hormone exposure and the risk for major malformations. JAMA 255(22): 3128-3132.

Laurence M, Miller M, Evans K, Carter C (1971) Hormonal pregnancy tests and neural tube malformations. Nature 233: 495-496

Levy EP,  Cohen A, Fraser FC (1973) Hormone treatment during pregnancy and congenital heart defects. Lancet 1(7803): 611

Nora JJA, Nora H, Blu J, Ingram J, Fountain A, Peterson M, Lortscher RH, Kimberling WJ (1978) Exogenous progestogen and estrogen implicated in birth defects. JAMA 240(9): 837-843

Polednak AP, Janerich DT (1983) Maternal characteristics and hypospadias: a case-control study. Teratology 28(1):67-73

Rothman KJ, Fyler DC, Goldblatt A, Kreidberg MB (1979) Exogenous hormones and other drug exposures of children with congenital heart disease. Am J Epidemiol 109(4): 433-439

Sainz MP, Rodriguez Pinilla E, Martinez-Frias ML (1987) Progestogens and estrogens in high doses (hormone pregnancy tests): the risk of appearance of spina bifida with anencephaly. Medicina clinica (Barcelona) 89: 272-274

Nora AH, Nora JJ (1975) A syndrome of multiple congenital anomalies associated with teratogenic exposure. Arch Envir Heatth. Vol 30

Janerich DT, Dugan JM, Standfast SJ, Strite L (1977) Congenital heart disease and prenatal exposure to exogenous sex hormones. BMJ 1(6068):1058-60

Tümmler G, Rißmann A, Meister R, Schaefer C (2014) Congenital bladder exstrophy associated with Duogynon hormonal pregnancy tests-signal for teratogenicity or consumer report bias? Reprod Toxicol ;45:14-9

Fleming DM (1978) Abnormal outcome of pregnancy after exposure to sex hormones. Personal communication. (Provided by Bayer).

Goujard J, Rumeau-Rouquette C, Saurel-Cubizolles MJ (1979) Hormonal tests of pregnancy and congenital malformations. Journal de gynecologie obstetrique et biologie de la reproduction 8: 489-196

Hadjigeorgiou E,  Malamitsi-Puchner A, Lolis D, Lazarides P (1982) Cardiovascular birth defects and antenatal exposure to female sex hormones. Developmental pharmacology and therapeutics 5(1-2): 61-67

Haller J (1974) Hormontherapie wahrend der graviditat.Deutsches Arzteblatt 14: 1013-1015

Kullander S, Kallen B (1976) A prospective study of drugs and pregnancy. Acta Onstet Gynecol Scand 55: 221-224

Meire F, Vuylsteek K (1978) Apr 1;1(6116):856. Continued use of hormonal pregnancy tests. BMJ 1:856

Michaelis J, Michaelis H, Gluck E, Koller S (1983) Prospective study of suspected associations between certain drugs administered during early pregnancy and congenital malformations. Teratology 27: 57-64

Roussel (1968) GP Survey – An investigation into the effects of oral pregnancy tests on the incidence of CNS malformations. Unpublished.

Rumeau-Rouquette et al (1978) Malformations congenitales risques perinatales. L’institut National de la Sante et de la recherce medicale (INSERM)

Torfs CP, Milkovich L, Van den Berg BJ (1981) The relationship between hormonal pregnancy tests and congenital anomalies: A prospective study. Am J Epidemiology 113(5): 563-574
