# Supplementary material for: Oral hormone pregnancy tests and the risks of congenital malformations: a systematic review and meta-analysis
Source: F1000Res. 2019 Jan 29;7:1725. Originally published 2018 Oct 31. [Version 2] doi: 10.12688/f1000research.16758.2 (PMC6281024; doi:10.12688/f1000research.16758.2)

Web Appendix 4 : Funnel Plots of all Congenital Malformations and Congenital Heart disease

All Congenital Malformations

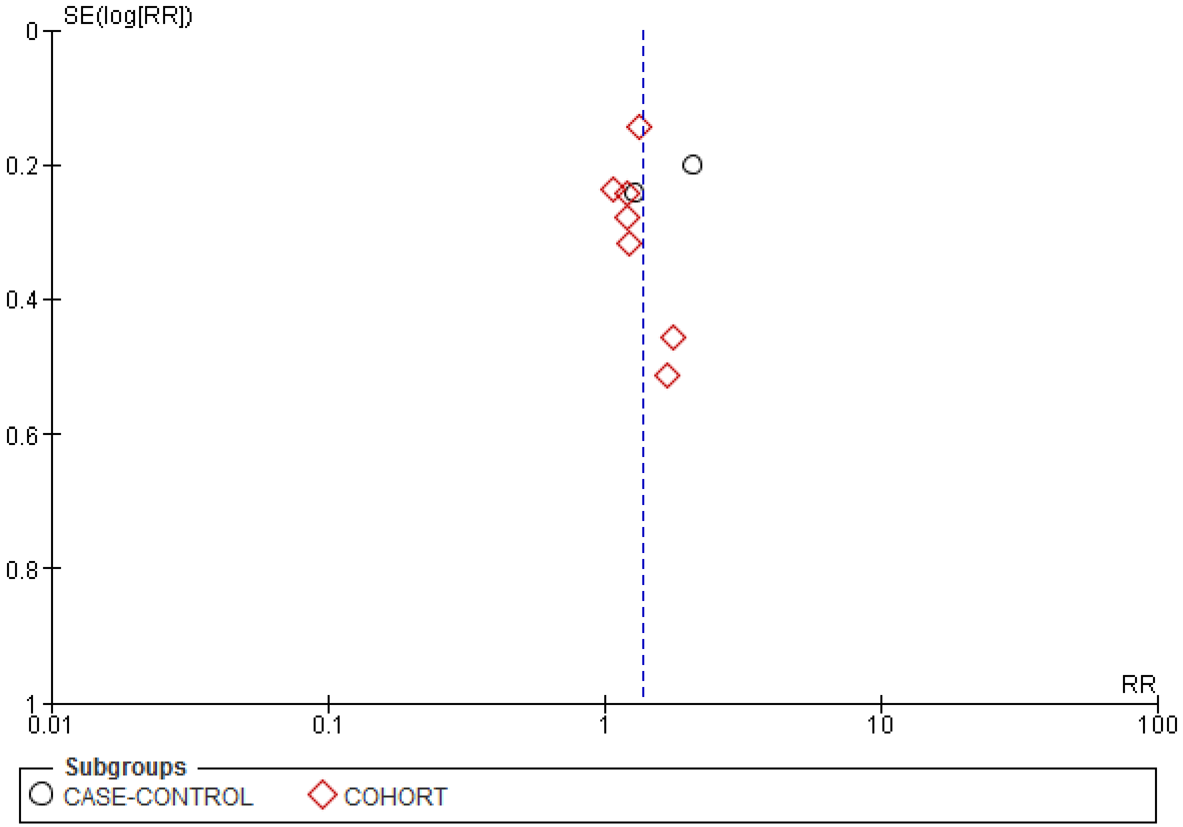

Congenital Heart Disease Malformations

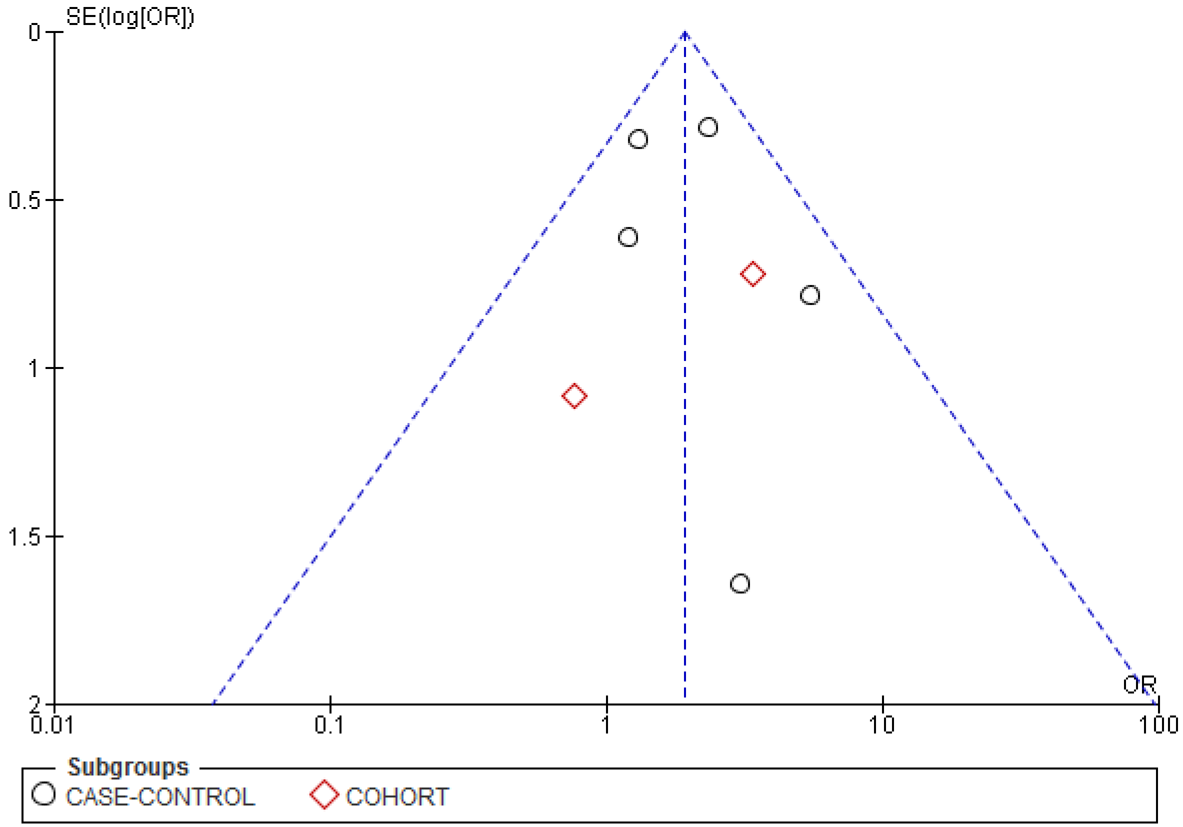

Supplement: Supplementary file 7 [file f1000research-7-19519-s0006.tgz › 4b5707b1-f734-4965-95bb-3f97414bd704_Supplementary_File_6_Funnel_plots.pdf]
