# Supplementary material for: Oral hormone pregnancy tests and the risks of congenital malformations: a systematic review and meta-analysis
Source: F1000Res. 2019 Jan 29;7:1725. Originally published 2018 Oct 31. [Version 2] doi: 10.12688/f1000research.16758.2 (PMC6281024; doi:10.12688/f1000research.16758.2)
Supplement: Peer review history

# Peer review: Referee response for version 1

**DOI:** 10.5256/f1000research.18318.r40887

**Published:** 2018-12-14

**Authors:** Olalekan A. Uthman

**Affiliations:** 1 Division of Health Sciences, Warwick Centre for Applied Health Research and Delivery, Warwick Medical School, University of Warwick, Coventry, UK

The manuscript reads well. The manuscript was well written. It has the potential to add to the body of knowledge in the field. The method was described in detail to allow for replication of the study. I have no major concerns.

 My only comment: the author should change the abstract conclusion to: “This systematic review and meta-analysis of **observational studies** shows that the use of oral HPTs in pregnancy is associated with increased risks of congenital malformations”.

I have read this submission. I believe that I have an appropriate level of expertise to confirm that it is of an acceptable scientific standard.

# Response

**Published:** 2019-01-16

**Authors:** Carl Heneghan

Many thanks for these positive comments. The methods and results are clear about the type of studies included, and we consider the conclusions do not require the addition of the study type. This is in line with previous systematic reviews our group has published.

# Peer review: Referee response for version 1

**DOI:** 10.5256/f1000research.18318.r40291

**Published:** 2018-11-29

**Authors:** Jesse Olszynko-Gryn, Cyrille Jean

**Affiliations:** 1 Department of History and Philosophy of Science, University of Cambridge, Cambridge, UK; 2 Sciences Po, Paris, France

This is a timely and much-needed paper that deserves to be widely read and cited. It provides the first systematic review and meta-analysis of old epidemiological data pointing towards a long-acknowledged association between HPTs and birth defects. Most of the paper is devoted to apparently rigorous statistical analysis. We leave constructive criticism of the statistics to other, more appropriately qualified reviewers. Instead, we confine our comments to the historical context and factual details presented in the paper. These, on the whole, are entirely satisfactory. But some minor errors — that do not significantly detract from the overall argument — should be amended:

  

1. 'Oral hormone pregnancy tests (HPTs), such as Primodos, containing ethinylestradiol and high doses of norethisterone, were given to over a million women from 1958 to 1978’ (p. 1). 
  It is worth clarifying that HPTs were available as injections from 1950 and in tablet form (e.g., Schering’s Orasecron, Roussel’s Amenorone Forte), in the UK, from at least 1956. See, for example, Britton (1956 ^1^); and https://archive.org/details/b19974760M4180/page/n45?q=amenorone+1956. For an extended discussion, see Olszynko-Gryn (2014), available for download here. Furthermore, not all HPTs contained norethisterone; different companies used other types of synthetic progesterone, and the same goes for ethinylestradiol.
2. ‘Oral hormone pregnancy tests (HPTs), such as Primodos (known as Duogynon in Germany), were used from 1958 to 1978, before urine pregnancy tests were available’ (p. 3).
  Contrary to popular belief, urine pregnancy tests were in fact widely though unevenly available between 1958 and 1978 and HPTs were never the dominant method of pregnancy testing. For a detailed timeline of pregnancy testing in the UK, please see Olszynko-Gryn *et al.* (2018 ^2^), esp. pp. 35-36. It would also be helpful to clarify that HPTs were removed from UK market in 1978, but earlier and later elsewhere. See Olszynko-Gryn *et al.* (2018 ^2^) for details (pp. 41-42).
3. ‘The test principle was that menstruation would be induced in those who were not pregnant’ (p.3).
  At the time HPTs were variously described as ‘clinical’, ‘hormonal’, or ‘withdrawal bleeding’ pregnancy tests and it would be more precise to refer to their effect as inducing *menstrual-like withdrawal bleeding*, which is not identical to menstruation.
4. Worth mentioning that Gal 1967, though a highly significant intervention, was not the first published warning against HPTs; these began to appear as early as 1956, in response to marketing literature aimed at GPs. See Britton (1956 ^1^) and Olszynko-Gryn *et al.* (2018 ^2^), p. 36.
5. ‘However, we believe that this systematic review shows an association of oral HPTs with congenital malformations’ (p. 17).
  More optionally, the authors might consider reflecting on the extent to which the association they identify implies a *causal *association. An association between the use of HPTs and birth defects has long been recognised and was rarely in dispute. Many experts explained the association in terms of a suspected though as unknown direct mechanistic effect of HPTs on the developing human embryo. Others, however, preferred to explain the association in terms of underlying factors, e.g., a patient history of miscarriage or birth defects. This view, which still has traction in some quarters, is discussed to some extent in Olszynko-Gryn *et al.* (2018 ^2^) (pp. 39-41). The authors might usefully offer a fresh perspective based on their findings, in the Conclusion and/or in the interesting discussion of unknown mechanisms on p. 16.

We have read this submission. We believe that we have an appropriate level of expertise to confirm that it is of an acceptable scientific standard.

## References

[1] BrittonH: Pregnancy Test. *BMJ*.1956;2(4989) : 10.1136/bmj.2.4989.419 10.1136/bmj.2.4989.419 13329365

[2] Olszynko-GrynJBjørvikEWeßelMJülichSJeanC: A historical argument for regulatory failure in the case of Primodos and other hormone pregnancy tests. *Reprod Biomed Soc Online*.2018;6: 10.1016/j.rbms.2018.09.003 34-44 10.1016/j.rbms.2018.09.003 30456319PMC6234516

# Response

**Published:** 2019-01-16

**Authors:** Carl Heneghan

Many thanks for these positive comments. 

We have amended the introduction with the following text: 'Oral hormone pregnancy tests (HPTs), such as Primodos (known as Duogynon in Germany), were available as injections from 1950 and in tablet form in the UK from 1956 onwards, before the modern forms of urine pregnancy tests became available [1]’

We have cited: Olszynko-Gryn J, Bjørvik E, Weßel M, Jülich S, Jean C. A historical argument for regulatory failure in the case of Primodos and other hormone pregnancy tests. Reprod Biomed Soc Online. 2018 Oct 23;6:34-44. Doi: 10.1016/j.rbms.2018.09.003. eCollection 2018 Aug.

We have amended the introduction text as per the reviewer's suggestion :

"Warnings about HPTs in pregnancy first emerged in 1956: accumulating concerns over an increased risk of malformations led to their withdrawal in a number of countries at different times. Norway cancelled the indication in pregnancy for HPTs in 1970; the UK did so in 1978, when the manufacturers of Primodos, Schering AG (taken over by Bayer AG in 2008), voluntarily stopped marketing the product; in Germany, Duogynon was taken off the market in 1981  [ref Olszynko-Gryn J].'

We have amended the introduction text as per the reviewer's suggestion:

‘The test principle was that they would induce bleeding similar to menstruation in those who were not pregnant.’

'Warnings about HPTs in pregnancy first emerged in 1956.'

And referenced the Britton H.G. Pregnancy test. Br. Med. J. 1956;2(18 Aug.):419. paper 

The benefits of our systematic review include that it quantifies the magnitude of the association and tests the robustness of this association across multiple studies by meta-analysis. We, therefore, perceive that they are rationale and the objectives are clear.

# Peer review: Referee response for version 1

**DOI:** 10.5256/f1000research.18318.r40053

**Published:** 2018-11-05

**Authors:** David Healy

**Affiliations:** 1 School of Medical Sciences, Bangor University, Bangor, UK

I am fully supportive of this article on the effects of hormone pregnancy tests as it stands. I have no substantive criticism of the content or methods.

 I am of course interested in why the regulator (MHRA) did not find comparable results but this is not a matter that should be addressed in this article.

 There is one extra point that this article may speak to which is that from some time it was thought that teratogens caused signature defects - such as the phocomelia of thalidomide. This may now be a minority position (I'm not sure of this point). The findings here do not support that point of view. I can understand if the authors may think that commenting on this point is a matter for others or for another article; I mention it for consideration.

 I have one very minor point about the layout which is that in the column where the numbers of women recruited to various studies is mentioned, the right justification of paragraphs leads to an odd spacing between 28 thousand and 671 - this doesn't apply when the page is resized.

I have read this submission. I believe that I have an appropriate level of expertise to confirm that it is of an acceptable scientific standard.

# Response

**Published:** 2019-01-16

**Authors:** Carl Heneghan

Many thanks for these positive comments.

Signature defects are only likely to occur if the timing of exposure is the same in all cases. However, when the timing of exposure varies, fetuses will be affected in different ways, depending on the tissues that are developing at the time of exposure, giving rise to a variety of malformations. The large range of times of exposure during embryogenesis determines which developmental processes are most affected, resulting in a wide variety of potential defects as seen in our review and in alleged Primodos survivors. Furthermore, different fetuses may have different epigenetic susceptibilities to different teratogenic outcomes.

The Primodos components norethisterone acetate and ethinyl estradiol induce developmental abnormalities in zebrafish embryos [Brown S, Fraga LR, Cameron G, Erskine L, Vargesson N. Sci Rep. 2018 Feb 13;8(1):2917. doi: 10.1038/s41598-018-21318-9]. Brown's data in Zebrafish show acetate and ethinylestradiol teratogenicity depends on dose and the embryonic stage of development, embryos at an early stage being more sensitive than those at a later stage.

The comments are interested in why the regulator (MHRA) did not find comparable results but this is not a matter that should be addressed in this article. We agree with this issue - no change required.

We have removed the spacing between numbers to eliminate the odd spacing effect.
